# Donor-transmitted cancer in kidney transplant recipients: a systematic review

**DOI:** 10.1007/s40620-020-00775-4

**Published:** 2020-06-13

**Authors:** Albino Eccher, Ilaria Girolami, Jennifer Danielle Motter, Stefano Marletta, Giovanni Gambaro, Rostand Emmanuel Nguefuet Momo, Francesco Nacchia, Paola Donato, Luigino Boschiero, Ugo Boggi, Letizia Lombardini, Massimo Cardillo, Antonietta D’Errico, Desley Neil, Dorry Lidor Segev, Gianluigi Zaza

**Affiliations:** 1 Pathology Unit, Department of Pathology and Diagnostics, University and Hospital Trust of Verona, P.le Stefani n. 1, 37126 Verona, Italy; 2 Department of Surgery, Johns Hopkins University School of Medicine, Baltimore, MA USA; 3 Renal Unit, University and Hospital Trust of Verona, Verona, Italy; 4 Department of Surgical Sciences, Kidney Transplant Center, University and Hospital Trust of Verona, Verona, Italy; 5 Division of General and Transplant Surgery, University of Pisa, Pisa, Italy; 6 National Transplant Center, Italian National Institute of Health, Rome, Italy; 7 Pathology Unit, S. Orsola-Malpighi University Hospital of Bologna, Bologna, Italy; 8 Department of Histopathology, University Hospital Birmingham, National Health Service Foundation Trust, Birmingham, UK

**Keywords:** Kidney transplantation, Donor-transmitted cancer, Disease transmission, Systematic review

## Abstract

The transmission of cancer from a donor organ is a rare event but has important consequences. Aim of this systematic review was to summarize all the published evidence on cancer transmission in kidney recipients. We reviewed published case reports and series describing the outcome of recipients with donor-transmitted cancer until August 2019. A total of 128 papers were included, representing 234 recipients. The most common transmitted cancers were lymphoma (n = 48, 20.5%), renal cancer (42, 17.9%), melanoma (40, 17.1%), non-small cell lung cancer (n = 13, 5.6%), neuroendocrine cancers comprising small cell lung cancer (n = 11, 4.7%) and choriocarcinoma (n = 10, 4.3%). There was a relative lack of glioblastoma and gastrointestinal cancers with only 6 and 5 cases, respectively. Melanoma and lung cancer had the worst prognosis, with 5-years overall survival of 43% and 19%, respectively; while renal cell cancer and lymphomas had a favorable prognosis with 5-years overall survival of 93 and 63%, respectively. Metastasis of cancer outside the graft was the most important adverse prognostic factor. Overall reporting was good, but information on donors’ cause of death and investigations at procurement was often lacking. Epidemiology of transmitted cancer has evolved, thanks to screening with imaging and blood tests, as choriocarcinoma transmission have almost abolished, while melanoma and lymphoma are still difficult to detect and prevent.

**Electronic supplementary material:**

The online version of this article (10.1007/s40620-020-00775-4) contains supplementary material, which is available to authorized users.

## Introduction

For patients with end-stage kidney disease, the benefits of transplantation greatly exceed the risks, thus making transplantation the best therapeutic option. However, transplantation carries an unavoidable risk of transmission of malignant diseases, and this risk may be enhanced when organs from donors with history of or ongoing malignancy are recovered. Moreover, donors are getting older and this increases the risk of an undetected malignancy. Transmission of cancer from donor to recipient was first recognized more than 50 years ago and many reports of transmission events have been published. The first systematic study on the topic is relatively recent, with the study of Xiao et al. in 2013 [1]. Despite the low incidence of cancer transmission in large case series from donors with history of or ongoing malignancy [2–4], there is still some concern regarding the use of such donors, because of high morbidity and mortality in recipients in cases of transmission [5]. Transmission of donor-derived malignancy also occurs from donors with no history of malignancy, thus vigilance during donor assessment is required. International guidelines and recommendations [6–9] are mainly based on single case reports/case series over different eras with different/evolving donor and recipient management throughout [10].

Interpretation is made difficult due to inconsistent definitions for a transmission event. Donor-related cancer (DRC) can be classified in donor-transmitted cancer (DTC), when the malignancy is present or presumed in the graft at time of transplantation, or donor-derived cancer (DDC), when cancer is not expected to exist at time of transplantation but develops within donor cells after transplantation [11]. Even with this definition there remain cases where it is not possible to discriminate between the two groups. Due to the uncertainty, criteria for a proven, probable or possible transmission event are outlined in Ison et al. [11]. Interpretation of the literature is further compounded by significant variability in assessing and reporting across different transplant service areas [11].

The aim of this study was to systematically review all the published evidence on cases of donor-related cancer among kidney transplant recipients and to attempt to assess in a more detailed manner donor management and recipient factors to provide insight into what factors impact on risk of transmission.

## Methods

We conducted a systematic review according to standard methods and reporting in accordance with the appropriate guidelines, Preferred Reporting Items for Systematc Reviews and Meta-Analysis (PRISMA) [12] and Meta-Analysis of Observational Studies in Epidemiology (MOOSE) [13]. No specific protocol was registered on PROSPERO or other databases.

### Search strategy and databases

The databases Pubmed, Scopus and the Cochrane Library were systematically searched without language restrictions until August 2019 to identify any study documenting cancer transmission to kidney transplant recipient. The NOTIFY library, a public project endorsed by the Italian National Transplant Center (CNT) [14], was also searched. Full texts assessed for eligibility underwent also reference hand-searching to identify relevant articles potentially missed. The search strategies can be found in Appendix S1.

### Inclusion and exclusion criteria

Two investigators screened titles and abstracts and disagreement was resolved by consultation of a third reviewer. Any article documenting a donor-transmitted or donor-derived cancer in a kidney recipient according to the Disease Transmission Advisory Committee (DTAC) was included [11]. Exclusion criteria were: not involving a kidney recipient, the sole transmission of oncogenic viruses and the statement in the report that donor transmission could be excluded. Donor-derived tumors with no evidence of predisposing infective agent were included. Any type of study that contains data pertinent to a cancer transmission event was included. Full texts of the articles fulfilling initial screening criteria were acquired and reviewed.

### Data extraction

Two authors extracted data from the included studies following a standardized extraction form. Data extracted were: donors’ and recipients’ age and sex, type of cancer, site of cancer if localized to the graft or metastasizing, treatment of the recipient, prior cancer history in the donor, whether the donor was a multi-organ donor, whether and how the donor was evaluated, donor cause of death, methods of establishing donor origin of cancer, time to cancer diagnosis after transplantation, outcome of the recipient, time to death of the recipient from cancer diagnosis and whether death was due to transmitted cancer.

The primary outcomes were the overall survival of recipients after transmitted cancer diagnosis and the time to cancer diagnosis after transplantation. The secondary outcomes were the distribution of cancer types, the frequency of metastasizing malignancies and the management of recipients.

### Quality assessment

The quality of included studies was assessed by two authors and disagreements were resolved by consultation of the third reviewer according to a standardized checklist. The specific items were modified and tailored to the specific issues of a cancer transmission event. When the article presented more than one case, the checklist was applied to every single case description. The checklist comprised the exhaustive description of the data above mentioned to be extracted. Adequate follow-up time was defined as at least 6 months or until the recipient’s death, following the previous reporting [1].

### Data synthesis and statistics

A descriptive synthesis of demographic data, types and sites of malignancies, evaluation of the donor and recipient’s treatment was provided. Continuous measures were expressed as mean with standard deviation (SD), median and range, while dichotomous variables were expressed as numerical values and percentages.

Time-to-event curves were calculated using the Kaplan–Meier method for overall recipients and for the most frequently transmitted malignancies. Cox proportional hazard univariable and multivariable analysis was used to assess the impact of clinical variables on recipients’ survival. Statistical significance was set at 0.05. All analyses were performed with statistical software R 3.6.1 (R Foundation for Statistical Computing, Vienna, Austria) and Stata 15.1/MP for Linux (College Station, TX, USA: StataCorp LLC).

## Results

### Literature search

Of the 9289 publications retrieved after removal of duplicates, 8945 were excluded after title and abstract screening. The remaining 344 articles were assessed in full-text form. Of these, 128 articles were included, comprising 72 case reports (n = 72 cases), 50 case series (n = 132 cases) and 6 registries (n = 30 cases), with a total of 234 recipients. The flow of article screening is depicted in Appendix Figure S1.

### Quality appraisal of studies and cases

The quality of reporting was overall good, with greater than 70% of cases providing clear information on seven out of eleven items. Information on recipients’ outcome and follow-up was present in all cases, while information on time to cancer diagnosis was missing in 8.5% of cases. Clear reporting of demographic data was more frequent in recipients than donors (180, 76.9% vs 111, 47.4%). Information on donors’ evaluation at procurement and cause of death (37.2% and 31.6% of cases, respectively) were the least reported items. Quality appraisal is shown in Appendix Figure S2.

### Characteristics of donors

The demographic data of donors are summarized in Table 1. There were 187 donors, of which 18 (9.6%) were living donors and 87 (46.5%) were multi-organ donors. Of the multi-organ donors, in 64 (73.6%) the other kidney was used, with transmission of cancer in 41 (64.1%). The mean age of all donors was 48.3 (17.2) years. The most frequent cause of death was cerebral hemorrhage/hemorrhagic stroke, but in 90 (48.1%) it was not reported. Donors had a past history of cancer or an ongoing malignancy in 32 (17.1%) cases. Donors were evaluated with clinical exam and blood tests, without imaging studies, in 35 (18.7%) cases; with imaging studies and/or biopsy of suspicious lesions in 19 (10.2%) cases; while the information was lacking in 131 (70%) cases. Autopsy was performed in 30 donors, of which 23 of cases before 2000; while in 12 autopsy led to the discover of the tumor, of which half were represented by unsuspected lymphomas and melanomas.

**Table 1:** Demographic data of donors

	Age [mean(SD); median (range)]	Gender
Lymphoma = 38 (20)	37.7 (23.5); 30.5 (7–71)	M = 11 (29) F = 6 (16) NA = 21 (55)
Renal cell carcinoma = 37 (20)	54.8 (10.2); 56.5 (32–73)	M = 19 (51) F = 9 (24) NA = 9 (24)
Melanoma = 30 (16)	54.4 (12.3); 51.5 (42–73)	M = 5 (17) F = 7 (23) NA = 18 (60)
NSCLC = 12 (6)	47.7 (12); 51 (29–63)	M = 5 (42) F = 5 (42) NA = 2 (16)
Neuroendocrine = 8 (4)	59 (6.3); 59 (51–67)	M = 5 (63) F = 2 (25) NA = 1 (12)
Choriocarcinoma = 7 (4)	33.1 (7.3); 30 (26–47)	F = 7 (100)
Glioblastoma = 3 (2)	37 (7.1); 37 (32–42)	M = 2 (67) NA = 1 (33)
Breast cancer = 4 (2)	45.3 (6.8); 43 (40–53)	F = 4 (100)
Leukemia = 7 (4)	51.5 (25.2); 47.5 (19–81)	M = 1 (14) F = 4 (57) NA = 2 (29)
Other = 41 (22)	49.4 (17.6); 53 (0.2–73)	M = 21 (51) F = 14 (34) NA = 6 (15)
Total = 187 (100)	48.3 (17.2); 51.5 (0.2–81)	M = 69 (37) F = 58 (31) NA = 60 (32)

Apart from age, numbers represent absolute values with percentages in parentheses

*F* female, *M* male, *NA* not available, *NSCLC* non-small cell lung cancer, *SD* standard deviation

### Characteristics of recipients

The demographic data of recipients are summarized in Table 2. The mean age of overall recipients was 45.4 (15.5) years. The tumors were limited to the graft in 94 (40.2%) cases, metastastic in 116 (49.6%) and not specified in 24 (10.2%). The tumor was limited to the graft in 66.7% of lymphomas and renal carcinoma, while 82.5% of melanomas, 61.5% of lung cancer and 72.7% of neuroendocrine cancers had metastasized outside the graft. The most frequent treatment was removal of graft and return to dialysis (87, 37.2%), while in 61 (26.1%) nephrectomy was followed by chemotherapy, radiotherapy or immunotherapy. In one out of five cases (49, 20.9%) the information on recipient’s treatment was lacking.

**Table 2:** Demographic data of recipients

	Age [mean(SD); median (range)]	Gender
Lymphoma = 48 (21)	48 (15.1); 51 (14–69)	M = 22 (46) F = 18 (37) NA = 8 (17)
Renal cell carcinoma = 42 (18)	44.2 (16.8); 47.5 (9–69)	M = 27 (64) F = 12 (29) NA = 3 (7)
Melanoma = 40 (17)	47.8 (13.4); 47.5 (19–70)	M = 10 (25) F = 13 (33) NA = 17 (42)
NSCLC = 13 (6)	38.5 (10); 39 (18–53)	M = 10 (77) F = 2 (15) NA = 1 (8)
Neuroendocrine = 11 (5)	44.2 (11.3); 41 (25–64)	M = 5 (45) F = 4 (36) NA = 2 (18)
Choriocarcinoma = 10 (4)	29.9 (9.8); 27 (20–47)	M = 2 (20) F = 6 (60) NA = 2 (20)
Glioblastoma = 6 (3)	31.5 (11.8); 27.5 (23–48)	M = 2 (33) F = 2 (33) NA = 2 (33)
Breast cancer = 5 (2)	38 (19.7); 37.5 (15–62)	M = 3 (60) F = 2 (40)
Leukemia = 9 (4)	56.8 (17.4); 58 (21–77)	M = 2 (22) F = 4 (44) NA = 3 (33)
Other = 50 (21)	47.2 (15.3); 50 (1.42–71)	M = 26 (52) F = 17 (34) NA = 7 (14)
Total = 234 (100)	45.4 (15.5); 47 (1.42–77)	M = 109 (47) F = 80 (34) NA = 45 (19)

Apart from age, numbers represent absolute values with percentages in parentheses

*F* female, *M* male, *NA* not available, *NSCLC* non-small cell lung cancer, *SD* standard deviation

### Frequencies of malignancy

The most frequent cancer types were lymphoma (48, 20.5%), renal cancer (42, 17.9%), melanoma (40, 17.1%) and non-small cell lung cancer 13 (5.6%). There were 11 (4.7%) neuroendocrine tumors comprising 7 small cell lung cancer and 4 from other sites not otherwise specified and 10 (4.3%) choriocarcinomas. Nine (3.8%) recipients developed leukemia, six (2.6%) glioblastoma and five (2.1%) breast cancer. A summary of data of donors and recipients is presented in Tables 3, 4 and 5. Full list of cancer with less than 5 cases is found in Appendix 2.

**Table 3:** Characteristics of donor and recipients with most common cancers

	Donors		Recipients	
Lymphoma	38 (20)		48 (21)	
	Donor type	D = 18 (47) L = 3 (8) NA = 17 (45)	Localization	Graft = 32 (66) Metastasizing = 9 (19) NA = 7 (15)
	Cause of death	Cerebral hemorrhage = 3 (8) Head trauma = 7 (18) Other = 3 (8) NA = 25 (66)	Treatment	Nx only = 8 (17) Nx-RTx = 1 (2) Nx-other = 13 (27) CHT-RT-IT only = 1 (2) NA = 25 (52)
	Donor study	Yes, with imaging or biopsy = 3 (8) Yes, without imaging = 3 (8) NA = 32 (84)	Immunosuppression	Withdrawal = 18 (38) NA = 30 (62)
	Autopsy was performed in 3 cases; 2 donors with cancer history			
Renal cell carcinoma	37 (20)		42 (18)	
	Donor type	D = 27 (73) L = 8 (22) NA = 2 (5)	Localization	Graft = 28 (67) Metastasizing = 11 (26) NA = 3 (7)
	Cause of death	Cerebral hemorrhage = 11 (30) Head trauma = 3 (8) NA = 23 (62)	Treatment	Nx only = 31 (74) Nx-RTx = 1 (2) Nx-other = 5 (11) CHT-RT-IT only = 1 (2) No/supportive = 3 (7) NA = 1 (2)
	Donor study	Yes, with imaging or biopsy = 6 (16) Yes, without imaging = 2 (5) NA = 29 (79)	Immunosuppression	Withdrawal = 11 (26) Mantained = 11 (26) NA = 20 (48)
	Autopsy was performed in 2 cases; 1 donor with cancer history			
Melanoma	30 (16)		40 (17)	
	Donor type	D = 28 (93) L = 2 (7)	Localization	Graft = 5 (12) Metastasizing = 33 (83) NA = 2 (5)
	Cause of death	Cerebral hemorrhage = 13 (43) Head trauma = 1 (3) CNS malignancy = 2 (7) Other malignancy = 1 (3) NA = 23 (62)	Treatment	Nx only = 12 (30) Nx-RTx = 1 (2) Nx-other = 15 (38) No/supportive = 9 (23) NA = 3 (7)
	Donor study	None = 2 (7) Yes, with imaging or biopsy = 1 (3) Yes, without imaging = 8 (27) NA = 19 (63)	Immunosuppression	Withdrawal = 23 (58) Mantained = 1 (2) NA = 16 (40)
	Autopsy was performed in 6 cases; 8 donors with cancer history			
Lung cancer	12 (6)		13 (6)	
	Donor type	D = 11 (92) NA = 1 (8)	Localization	Graft = 4 (31) Metastasizing = 8 (61) NA = 1 (8)
	Cause of death	Head trauma = 1 (8) Other malignancy = 2 (17) Other = 3 (25) NA = 6 (50)	Treatment	Nx only = 5 (39) Nx-other = 1 (8) CHT-RT-IT only = 2 (15) No/supportive = 3 (23) NA = 2 (15)
	Donor study	Yes, without imaging = 7 (58) NA = 5 (42)	Immunosuppression	Withdrawal = 4 (31) Mantained = 1 (8) NA = 8 (61)
	Autopsy was performed in 7 cases; 5 donors with cancer history			
Breast cancer	4 (2)		5 (2)	
	Donor type	D = 3 (75) L = 1 (25)	Localization	Graft = 2 (40) Metastasizing = 3 (60)
	Cause of death	Cerebral hemorrhage = 1 (25) Other malignancy = 1 (25) Other = 1 (25) NA = 1 (25)	Treatment	Nx-other = 1 (20) CHT-RT-IT only = 1 (20) No/supportive = 3 (60)
	Donor study	Yes, with imaging or biopsy = 1 (25) Yes, without imaging = 2 (50) NA = 1 (25)	Immunosuppression	Withdrawal = 1 (20) Mantained = 1 (20) NA = 3 (60)
	Autopsy was performed in 2 cases; 2 donors with cancer history			

*CHT* chemotherapy, *CNS* central nervous system, *D* deceased donor, *F* female, *IT* immunotherapy, *L*, living donor, *M* male, *NA* not available, *Nx* explant nephrectomy, *RT* radiotherapy, *RTx* retransplant, *SD* standard deviation. All data are in absolute number and percentage in parentheses

**Table 4:** Characteristics of donors and recipients with glioblastoma

Donors = 3	
Age [mean (SD), median (range)]	37 (7.1); 37 (32-42)
Gender	M = 2 (67) NA = 1 (33)
Donor type	D = 3 (100)
Cause of death	CNS malignancy = 1 (33) NA = 2 (67)
Donor study	NA = 3 (100)
	No autopsies were performed; in all donors history of glioblastoma was known
Recipients = 6	
Age (mean (SD), median (range))	31.5 (11.8); 27.5 (23-48)
Gender	M = 2 (33) F = 2 (33) NA = 2 (33)
Localization	Graft only = 4 (67) Metastasizing = 2 (33)
Treatment	Nx only = 5 (83) Nx-other = 1 (17)
Immunosuppression	Withdrawal = 4 (67) NA = 2 (33)
	Available follow-up was 10–36 months; one of the recipients with metastasizing disease died

All data apart from age are in absolute number and percentage in parentheses

*CNS* central nervous system, *D* deceased donor, *F* female, *M* male, *NA* not available, *Nx* explants nephrectomy, *SD* standard deviation

**Table 5:** Characteristics of donors and recipients of less frequent cancers

	Donors		Recipients	
Neuroendocrine	8 (4)		11 (5)	
	Donor type	D = 6 (75) L = 2 (25)	Localization	Graft = 3 (27) Metastasizing = 8 (73)
	Cause of death	Cerebral hemorrhage = 3 (38) NA = 5 (62)	Treatment	Nx only = 3 (27) Nx-other = 8 (73)
	Donor study	Yes, with imaging or biopsy = 3 (38) NA = 5 (62)	Immunosuppression	Withdrawal = 5 (45) NA = 6 (55)
Choriocarcinoma	7 (4)		10 (4)	
	Donor type	D = 7 (100)	Localization	Graft = 2 (20) Metastasizing = 6 (60) NA = 2 (20)
	Cause of death	Cerebral hemorrhage = 7 (100)	Treatment	Nx only = 2 (20) Nx-other = 6 (60) CHT-RT-IT only = 2 (20)
	Donor study	Yes, with imaging or biopsy = 3 (43) Yes, without imaging = 1 (14) NA = 3 (43)	Immunosuppression	Withdrawal = 7 (70) NA = 3 (30)
	Autopsy was performed in 2 donors			
Leukemia	7 (4)		9 (4)	
	Donor type	D = 6 (86) NA = 1 (14)	Localization	Graft = 3 (33) Metastasizing = 4 (44) NA = 2 (22)
	Cause of death	Cerebral hemorrhage = 4 (57) Other = 1 (14) NA = 2 (29)	Treatment	Nx-other = 2 (22) CHT-RT-IT only = 5 (56) NA = 2 (22)
	Donor study	Yes, without imaging = 3 (43) NA = 4 (57)	Immunosuppression	Withdrawal = 4 (44) Mantained = 1 (11) NA = 4 (44)
	Autopsy was performed in 2 donors			
Other	41 (22)		50 (21)	
	Donor type	D = 38 (93) L = 2 (5) NA = 1 (2)	Localization	Graft = 11 (22) Metastasizing = 32 (64) NA = 7 (14)
	Cause of death	Cerebral hemorrhage = 14 (34) Head trauma = 2 (5) CNS malignancy = 3 (7) Other malignancy = 3 (7) Other = 13 (32) NA = 6 (15)	Treatment	Nx only = 21 (42) Nx-other = 15 (30) CHT-RT-IT only = 3 (6) No/supportive = 7 (14) NA = 4 (8)
	Donor study	Yes, with imaging or biopsy = 2 (5) Yes, without imaging = 9 (22) NA = 30 (73)	Immunosuppression	Withdrawal = 31 (62) Mantained = 1 (2) NA = 18 (36)
	Autopsy was performed in 7 donors; 10 donors with history of cancer			

*CHT* chemotherapy, *CNS* central nervous system, *D* deceased donor, *F* female, *IT* immunotherapy, *L* living donor, *M* male, *NA* not available, *Nx* explant nephrectomy, *RT* radiotherapy, *RTx* retransplant, *SD* standard deviation. All data are in absolute number and percentage in parentheses

### Outcome of overall recipients

**Fig. 1:**
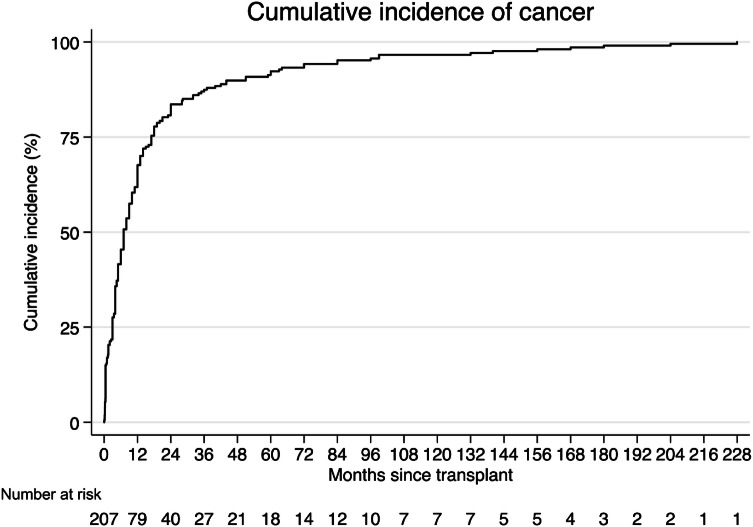
Time to cancer diagnosis from transplantation for all recipients

The time to cancer diagnosis is shown in Fig. 1. The median time to cancer diagnosis was 7 months (IQR 3–17) and the diagnosis had been made in 68% and 84% recipients at 1 year and 2 years post-transplant respectively. Localized cancers were diagnosed earlier than metastasizing cancers (log-rank test, p = 0.03).

**Fig. 2:**
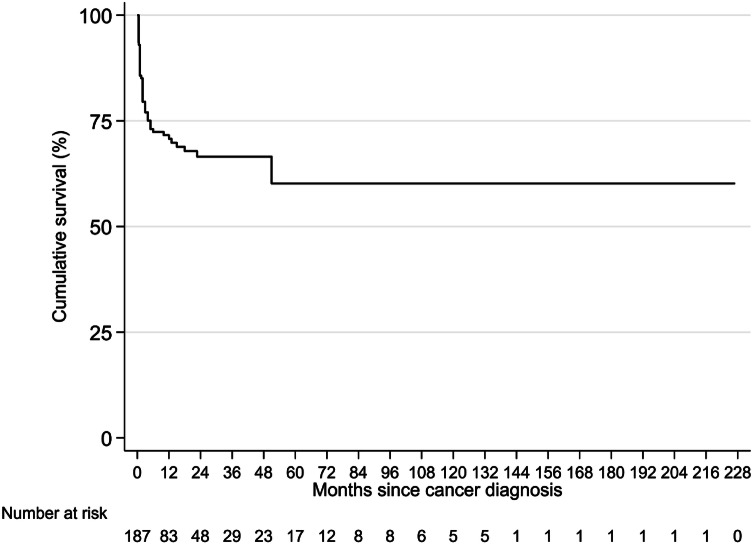
Overall survival for all recipients

The overall survival after cancer diagnosis is shown in Fig. 2. Cancers that had metastasized at the time of diagnosis had a worse overall survival (log-rank test, p < 0.001). In recipients in which the tumor had metastasized, nephrectomy and other therapy led to a better survival than surgery alone or systemic therapy alone (overall log-rank test, p < 0.001). In univariable regression analysis, tumor metastasis was the strongest negative prognostic factor (HR 40.05, CI 5.51–290.96, p < 0.001). Treatment with systemic therapy alone or supportive treatment were associated with worse prognosis (HR 2.56, CI 1.02–6.43, p = 0.05 and HR 4.13, CI 1.75–9.72, p = 0.001 respectively) and female sex was associated with a slightly worse survival (HR 1.76, CI 0.96-3.22, p = 0.07). In multivariable regression analysis tumor metastasis retained a strong adverse prognostic value (HR 31.71, CI 4.27–235.52, p = 0.001), followed by supportive treatment only (HR 4.73, CI 1.66–13.47, p = 0.004).

### Subgroups of malignancy

**Fig. 3:**
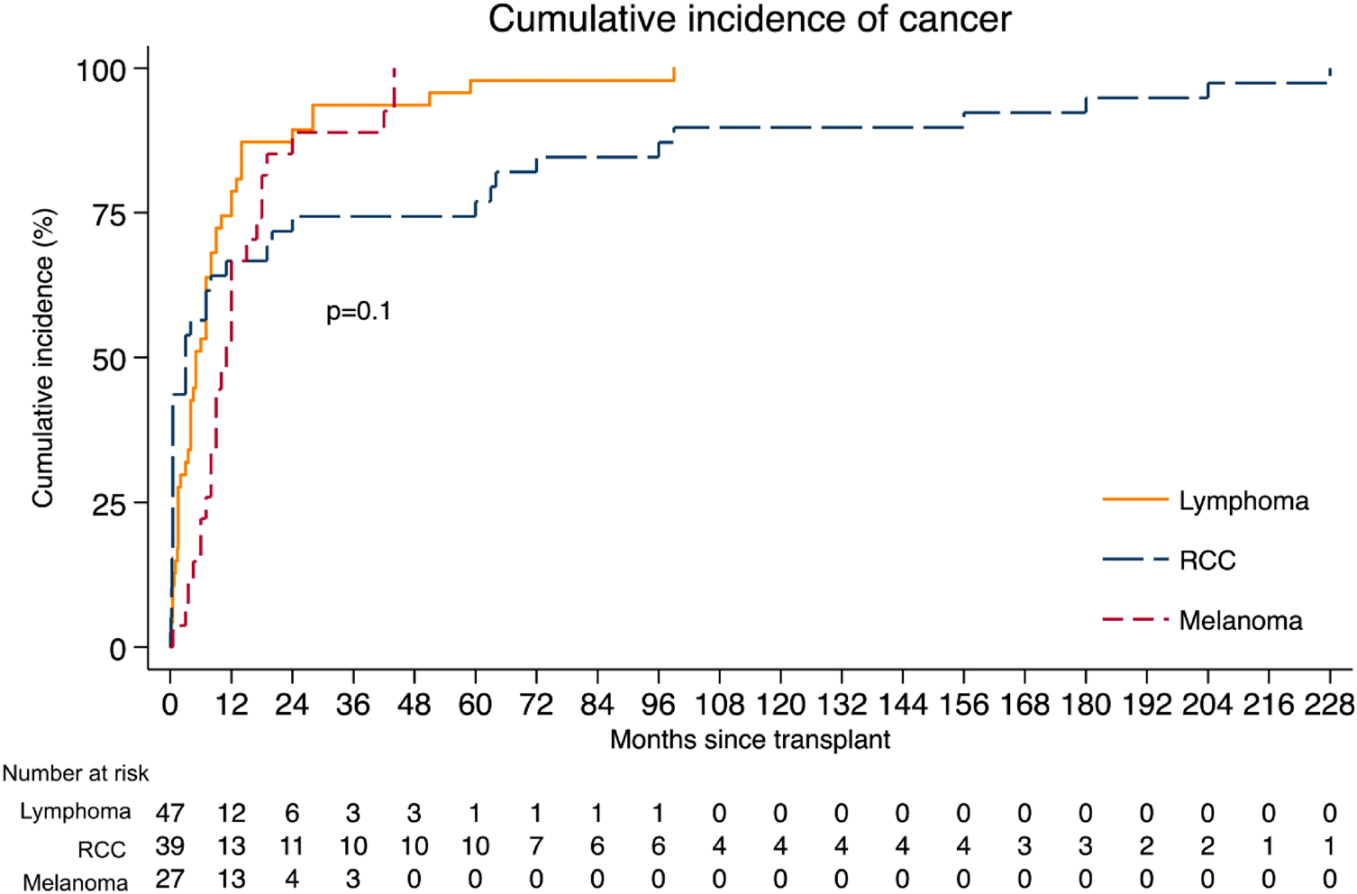
Time to cancer diagnosis from transplantation for most frequent cancers

The time to cancer diagnosis for the three most frequent cancer types is shown in Fig. 3. Median time to diagnosis for lymphoma was 5 months (IQR 1.5–12), for renal carcinoma was 3 months (IQR 0.3–60) and for melanoma was 11 months (IQR 7–18). For the other most represented tumors, median time to diagnosis was 13 months (IQR 6–17) for NSC lung cancer, 10 months (IQR 7–12) for neuroendocrine neoplasms and 2.3 months (IQR 1–3) for choriocarcinoma.

**Fig. 4:**
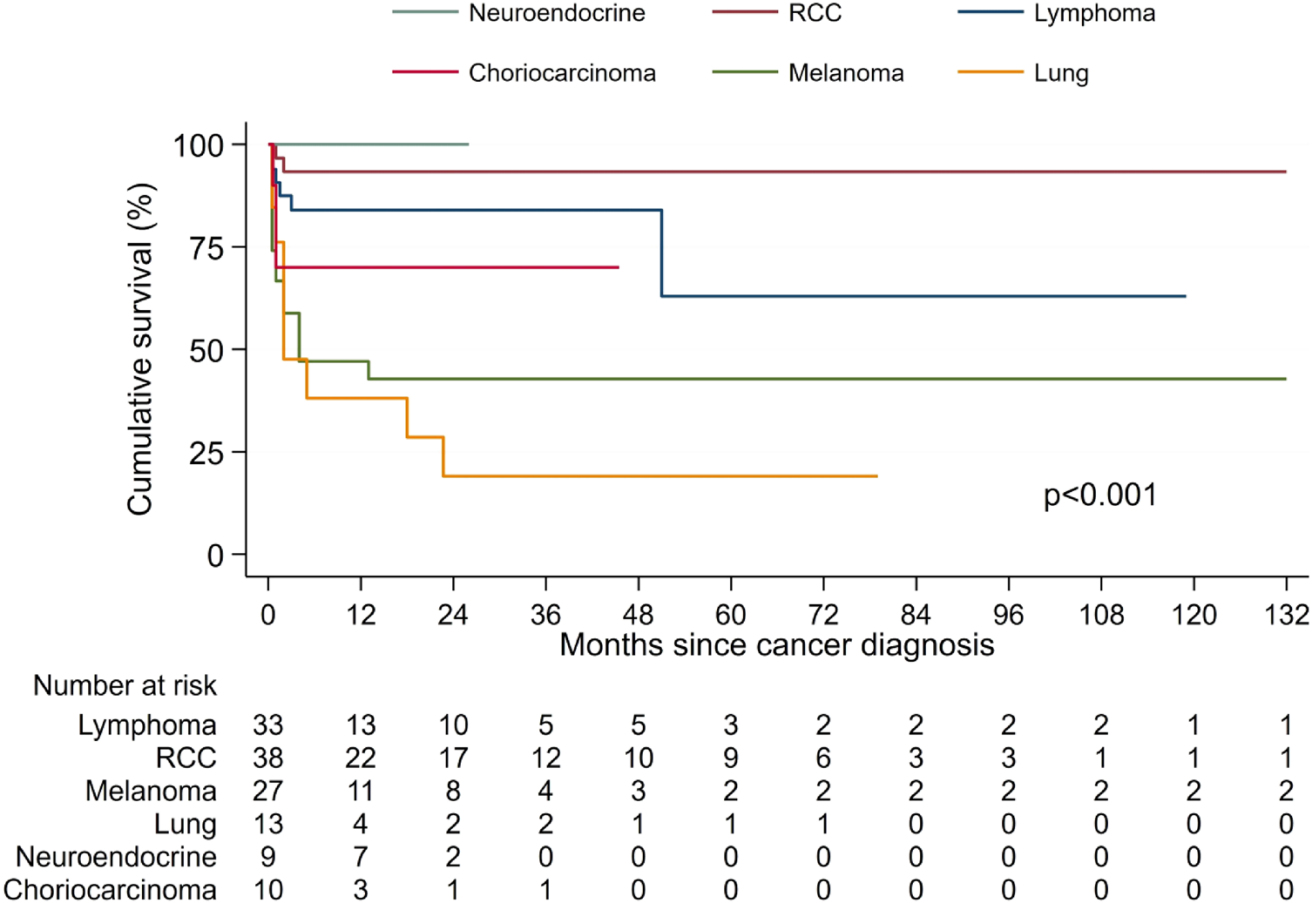
Overall survival for recipients with most frequent cancers

The overall survival for the most frequent cancers is shown in Fig. 4. Melanoma and NSC lung cancer had the worst prognosis, with median survival of 4 and 2 months after cancer diagnosis, respectively. Overall survival at 2-year and 5-year was 43% for melanomas and 19% for lung cancer. Melanomas limited to the graft were all alive at the end of their follow-up, while metastatic melanomas had a 5-years survival of 33% (log-rank test, p = 0.03). The subset of recipients with metastatic melanoma treated with nephrectomy and additional systemic therapy had a better survival than recipients treated by transplant nephrectomy alone or supportive therapy only (overall log-rank test, p = 0.002). For NSC lung cancer, there was no significant difference between localized and metastatic tumors (log-rank test, p = 0.17). Renal cell cancer and lymphoma showed better prognosis, with 93% and 63% overall survival at 5-years. Renal cell cancers limited to the graft were all alive at 5 years and they were all treated with excision of the lesion, removal of graft or only follow-up in some cases. All the lymphomas localized to the graft were alive at 5 years, while only 60% of lymphomas that had spread were alive at 5 years (log-rank test, p = 0.002). Lymphoma recipients were treated mainly by removal of graft with or without additional chemotherapy, but without significant difference in survival according to treatment (overall log-rank test, p = 0.94).The recipients with neuroendocrine tumors were all alive at the end of their known follow-up and they were all treated with removal of graft with or without additional chemotherapy.

## Discussion

Information provided in published case studies, series and registries contained all relevant information in only one-third of cases. The major area where data was lacking was the imaging investigations undertaken in the donor assessment.

Improvements in chemotherapy regimens, together with the possibility of transplant nephrectomy and returning to renal replacement therapy, have improved the prognosis of recipients with donor-transmitted cancer. Donors dying with malignancy may still be suitable donors and histological assessment of newly discovered lesions, even if they turn out to be neoplastic may be suitable to transplantation because of the low risk of transmission. This may apply also to lymphoproliferative disease found limited to the graft after transplantation, while it remains of great importance to detect active ongoing lymphoma in the donor that can subsequently spread in the recipient [5]. It is extremely encouraging that the transmission of some cancers, in which there are specific donor guidelines to aid detection [9], namely choriocarcinoma, glioblastoma and NSC lung carcinoma have decreased. Indeed, all reported cases are dating back to the 1990s, before the establishment of guidelines and a critical evaluation of occurred cases [9]. Transmissions of melanomas and lymphomas are more evenly distributed over years, reflecting potential difficulties in detecting these tumors in the donor. Interestingly, there was a relative lack of gastrointestinal cancer transmission with only 5 cases, and this is important to note as gastrointestinal cancer is relatively common in general population.

The use of autopsies to assess the donors is decreasing likely due to the cost of performing an autopsy [15] and a reluctance of the donor’s family, together with the greater availability of imaging techniques. However, autopsy can lead to the discovering of an unsuspected cancer, particularly for malignancies difficult to detect [16, 17]. A definitive diagnosis of malignancy may not be possible immediately with frozen section and in most instances can be made within a few days however, allowing early and individualized management/treatment of the recipient, preventing the development of cancer. Every suspicious lesion should be investigated, because diagnosing a malignancy can drive the management of recipients, even in cases when a reliable diagnosis is achieved some days after [17, 18].

Care should be taken with potential donors dying of cerebral hemorrhage as this may be due to a bleed into an “unexpected” metastatic lesion. This presumably lead to the accidental transmission of choriocarcinoma following the death of pregnant female with a bleed from a presumed vascular malformation [19]. Apart from this particular case, transmission of choriocarcinoma is now virtually eliminated due to the use of β-HCG screening test, with all other cases having occurred 25 or more years ago [20–25].

Time to tumor transmission diagnosis has not been determined in previous reviews. Our results show that diagnosis of a transmitted cancer is usually made soon after the transplant, with 68% and 84% of recipients being diagnosed within 12 and 24 months. This finding suggests that vigilance and a high index of suspicion whilst managing recipients in the first 2 years after transplant allow the diagnosis to be made at the earliest time point.

Transmitted melanoma and NSC lung cancer have the worst survival outcome, with a 2-years overall survival after cancer diagnosis of 43 and 19% respectively in our series. The poor survival of these tumors is not unexpected, due to their high malignant potential. Survival rate for melanoma has improved [1], and is likely to improve further with the new targeted therapies [26]. Unlike most other tumor types a prolonged recurrence free period does not lessen/remove the risk of transmission, with documented transmission occurring from a donor more than 16 years after the original diagnosis [27]. Most transmitted melanomas come from donors with no known history at the time of transplantation. After diagnosis in the recipient, cases of “forgotten” history are identified [27–29]. Anyway, a donor history of melanoma however does not always result in transmission to the recipient [28]. From the analysis of the literature, pathological data on melanoma features are not recorded. Thus it is not possible to identify any criteria of when a history of melanoma in the donor predicts the risk of recurrence in the recipient [29]. More detailed data collection would likely give a better understanding and improve risk stratification.

The majority of transmitted NSC lung cancer is from more than 30 years ago and this may be due to the awareness of the malignant potential, resulting in improved donor evaluation. There are however still instances of transmission of NSC lung cancer identified from registries and as unfortunately the management of the donor is not described, it is impossible to learn anything new.

Recipients with donor-transmitted renal carcinoma have the best prognosis, with a 5-years overall survival of 93%. These findings are in line with present literature, supporting the use of donors with renal cancer with a reasonable degree of safety [1, 6, 9, 30]. Most of these tumors are identified when they are restricted to the graft in the first year post-transplant, possibly because of imaging performed for the evaluation of graft function. Because of the localization to the graft they were treated predominantly by transplant nephrectomy, resection of the tumor, and in some cases by follow-up only. The cases with adverse outcome showed histological sarcomatoid features [31], or were historical poorly described reports [32]. Of those diagnosed late post-transplant only a few are of proven donor origin. It is possible that only those diagnosed in the early post-transplant period are strictly speaking DTC, being present but unrecognized at time of transplantation and then increased in size. Those diagnosed late post-transplant may be better defined as donor-derived. For the purposes of this study to take into account of the uncertainty in definitions [11] all the cases of proven donor origin or where there is a suspicion of transmission according to the definitions of DTAC are included.

Kidney recipients, because of the option of return to dialysis, can be treated maximally if a transmitted cancer is found. They can thus undergo withdrawal of immunosuppression, removal of the graft and chemotherapy. This may explain the favorable outcome of transmitted neuroendocrine cancers, which were all treated with nephrectomy, with the addition of chemotherapy in the metastasizing cases. However, none of the neuroendocrine tumor were discovered or suspected before transplantation. Even if removal of graft and chemotherapy seem to be effective, there is the need to prevent the burden of morbidity due to the transmission of such cancer. Almost the same apply to lymphomas, where no donor had history or evidence of lymphoproliferative disease. However, they are unlikely to be discovered in the time constraints before transplantation, as a neoplastic clone that could be present could not be evident with routine donor evaluation [16, 33]. As already stated elsewhere, there is still ongoing controversy regarding whether lymphoma should be defined DDC or DTC [1], with the majority of post-transplant lymphoproliferative disease of recipient origin, often after reactivation of previously acquired EBV infection [34]. All the cases included in this study were of proven donor origin, with more than 70% of cases confirmed with molecular techniques and the other with epidemiological criteria. More than half of these tumors were limited to the graft once developing in the recipient, in contrast with what more frequently encountered in lymphoproliferative disorders of host origin [34].

The limitations of this study reside in the nature of the primary studies, which comprise mainly case reports of different eras, and only few registries and large series. This could have hampered the precise estimation of cancer-specific outcome. Highly variable follow-up times, inconsistent detail of reporting of donor history, diagnosis and evaluation, differences in treatment may have precluded a reliable estimation of the role of these potential confounders in affecting the outcome and in stratifying the risk of specific cancer. Moreover, missing information for such diverse publications could hardly be imputed. There thus remains ongoing difficulties in obtaining accurate information on which to base the development of guidelines for risk stratification. Improved and continually updating international databases, with methods to increase complete minimum dataset entry are required to allow accurate guidelines to be developed and refined based on regular systematic review of the data every time important changes are made in treatment regimens, diagnostic procedures’ availability and changes in prognostic factors in the different cancer subtypes based on non-transplant outcomes.

## Electronic supplementary material

Below is the link to the electronic supplementary material.

Supplementary material 1 (DOCX 236 kb)

## Data Availability

Not applicable.
