# Supplementary material for: Donor-transmitted cancer in kidney transplant recipients: a systematic review
Source: J Nephrol. 2020 Jun 13;33(6):1321–32. doi: 10.1007/s40620-020-00775-4 (PMC7701067; doi:10.1007/s40620-020-00775-4)
Supplement: Supplementary file 1 — Supplementary material 1 (DOCX 236 kb) [file 40620_2020_775_MOESM1_ESM.docx]

**Supplementary material**

**Donor-transmitted cancer in kidney transplant recipients: a systematic review.**

Albino Eccher MD^1^*, Ilaria Girolami MD^1^*, Jennifer Danielle Motter MD^2^, Stefano Marletta MD^1^, Gianluigi Zaza MD^3^, Giovanni Gambaro MD^3^, Rostand Emmanuel Nguefuet Momo MD^4^, Francesco Nacchia MD^4^, Paola Donato MD^4^, Luigino Boschiero MD^4^, Ugo Boggi MD^5^, Letizia Lombardini MD^6^, Massimo Cardillo MD^6^, Antonietta D’Errico MD^7^, Desley Neil MD^8^, Dorry Lidor Segev MD^2^

^1^Pathology Unit, Department of Pathology and Diagnostics, University and Hospital Trust of Verona, Verona, Italy

^2^Department of Surgery, Johns Hopkins University School of Medicine, Baltimore, MA, USA

^3^Renal Unit, University and Hospital Trust of Verona, Verona, Italy

^4^Department of Surgical Sciences, Kidney Transplant Center, University and Hospital Trust of Verona, Verona, Italy

^5^Division of General and Transplant Surgery, University of Pisa, Pisa, Italy

^6^National Transplant Center, Italian National Institute of Health, Rome, Italy

^7^Pathology Unit, S. Orsola-Malpighi University Hospital of Bologna, Bologna, Italy

^8^Department of Histopathology, University Hospital Birmingham, National Health Service Foundation Trust, Birmingham, UK

*AE and IG should be considered joint first author

**Address correspondence and reprint requests to:** Albino Eccher, Department of Pathology and Diagnostics, University and Hospital Trust of Verona, P.le Stefani n. 1; 37126, Verona, Italy. Phone: +390458122161, Fax: +390458122011, e-mail: albino.eccher@aovr.veneto.it

**Appendix 1** – search strategies

Pubmed

(“tissue donor*” OR “living donor*” OR “organ donor*” OR “donor*” OR “graft” OR “allograft” OR “cadaver*” OR “recipient”) AND (“malignan*” OR “cancer” OR “neoplasm” OR “tumor” OR “tumour” OR “carcinoma” OR “adenocarcinoma” OR “lymphoma” OR “melanoma” OR “sarcoma”) AND (“transmission” OR “transmit*” OR “derived” OR “related”) AND “transplant*”

Scopus

TITLE-ABS-KEY ( *"donor*"*  OR  *"graft"*  OR  *"allograft"*  OR  *"cadaver*"*  OR  *"recipient"* )  AND  TITLE-ABS-KEY ( *"malignan*"*  OR  *"cancer"*  OR  *"neoplasm"*  OR  *"tumor"*  OR  *"tumour"* )  AND  TITLE-ABS-KEY ( *"transmission"*  OR  *"transmit*"*  OR  *"derived"*  OR  *"related"* )  AND  TITLE-ABS-KEY ( *"transplant*"* )  AND  ( EXCLUDE ( SUBJAREA ,  *"CENG"* )  OR  EXCLUDE ( SUBJAREA ,  *"ENGI"* )  OR  EXCLUDE ( SUBJAREA ,  *"AGRI"* )  OR  EXCLUDE ( SUBJAREA ,  *"NEUR"* )  OR  EXCLUDE ( SUBJAREA ,  *"MATE"* )  OR  EXCLUDE ( SUBJAREA ,  *"DENT"* )  OR  EXCLUDE ( SUBJAREA ,  *"CHEM"* )  OR  EXCLUDE ( SUBJAREA ,  *"PHYS"* )  OR  EXCLUDE ( SUBJAREA ,  *"COMP"* )  OR  EXCLUDE ( SUBJAREA ,  *"VETE"* )  OR  EXCLUDE ( SUBJAREA ,  *"ENVI"* )  OR  EXCLUDE ( SUBJAREA ,  *"ARTS"* )  OR  EXCLUDE ( SUBJAREA ,  *"SOCI"* )  OR  EXCLUDE ( SUBJAREA ,  *"MATH"* )  OR  EXCLUDE ( SUBJAREA ,  *"BUSI"* )  OR  EXCLUDE ( SUBJAREA ,  *"ENER"* ) )  AND  ( EXCLUDE ( EXACTKEYWORD ,  *"Hematopoietic Stem Cell Transplantation"* )  OR  EXCLUDE ( EXACTKEYWORD ,  *"Nonhuman"* )  OR  EXCLUDE ( EXACTKEYWORD ,  *"Animals"* )  OR  EXCLUDE ( EXACTKEYWORD ,  *"Mouse"* )  OR  EXCLUDE ( EXACTKEYWORD ,  *"Bone Marrow Transplantation"* )  OR  EXCLUDE ( EXACTKEYWORD ,  *"Mice"* )  OR  EXCLUDE ( EXACTKEYWORD ,  *"Animal"* )  OR  EXCLUDE ( EXACTKEYWORD ,  *"Animal Experiment"* )  OR  EXCLUDE ( EXACTKEYWORD ,  *"Allogeneic Hematopoietic Stem Cell Transplantation"* )  OR  EXCLUDE ( EXACTKEYWORD ,  *"Stem Cell Transplantation"* )  OR  EXCLUDE ( EXACTKEYWORD ,  *"Animal Model"* )  OR  EXCLUDE ( EXACTKEYWORD ,  *"Animal Cell"* )  OR  EXCLUDE ( EXACTKEYWORD ,  *"Animal Tissue"* )  OR  EXCLUDE ( EXACTKEYWORD ,  *"Allogeneic Stem Cell Transplantation"* )  OR  EXCLUDE ( EXACTKEYWORD ,  *"In Vitro Study"* )  OR  EXCLUDE ( EXACTKEYWORD ,  *"Allogenic Bone Marrow Transplantation"* )  OR  EXCLUDE ( EXACTKEYWORD ,  *"Rat"* ) )

CENTRAL

((“tissue donors” OR “living donors” OR donor selection) AND (kidney transplantation OR liver transplantation OR organ transplantation OR tissue transplantation OR transplantation)) AND ((donor NEAR/5 (malignan* OR cancer* OR tumo?r OR neoplasm*)) OR (transmission* OR transmit* NEAR/5 (donor* OR cancer* OR tumo?r* OR malignan* OR neoplasm*)))

Clinicaltrial.gov

(transplant OR transplantation) AND (transmission OR transmit) AND donor AND (cancer OR neoplasm OR malignancy OR tumour OR tumor)

**Appendix Figure S1.** Search flow diagram. The diagram has been realized according to the template of the PRISMA flow diagram available at the official PRISMA webpage (www.prisma-statement.org)


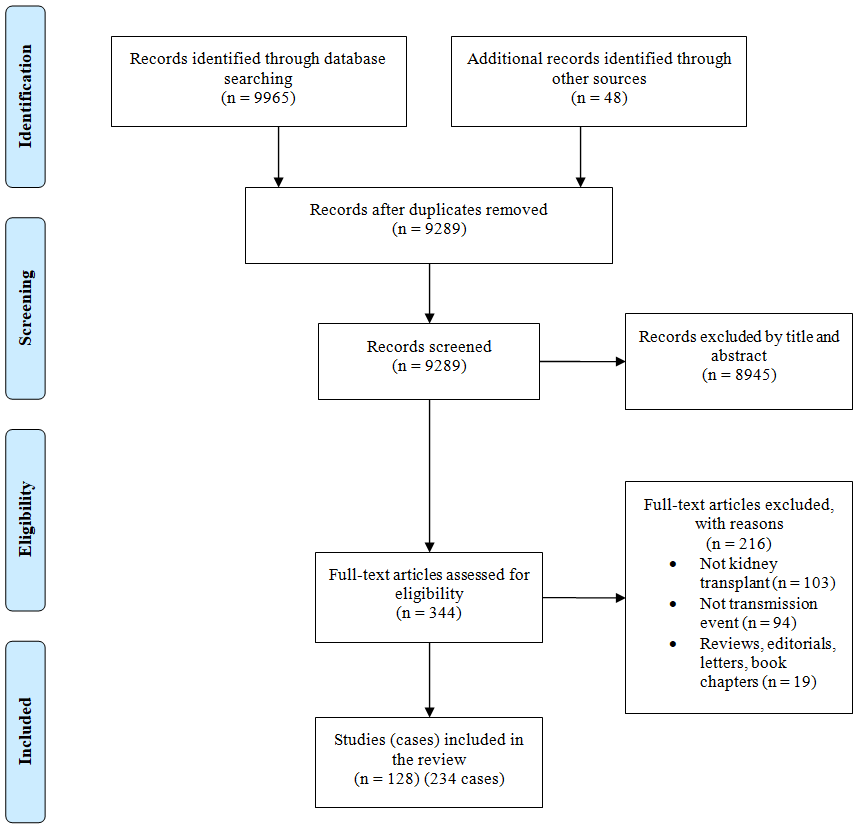


**Appendix Figure S2.** Quality appraisal.

**
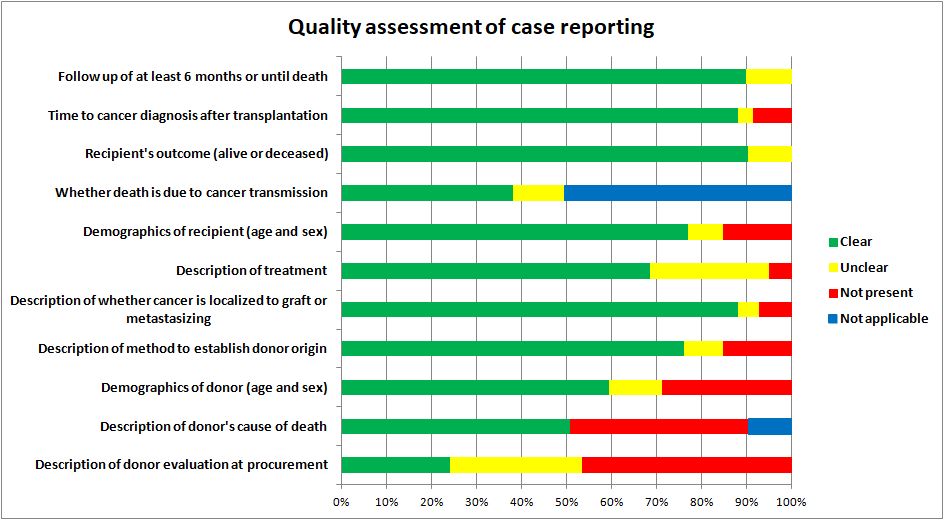
**

**Appendix 2** – Cancer types with less than 5 cases

| **Cancer types** | **Number of cases** |
| --- | --- |
| Adenocarcinoma, NOS | 2 |
| Adenocarcinoma, GI | 2 |
| Anaplastic carcinoma, NOS | 2 |
| Anaplastic thyroid carcinoma | 1 |
| Bile duct carcinoma | 1 |
| Clear cell adenocarcinoma | 1 |
| Germinal cell carcinoma | 2 |
| Hepatocellular carcinoma | 2 |
| Intestinal high-grade tumor, NOS | 2 |
| Kaposi sarcoma | 5 |
| Medulloblastoma | 2 |
| Meningioma | 2 |
| Myeloma | 4 |
| Ovarian cancer | 1 |
| Pancreatic adenocarcinoma | 1 |
| Papillary carcinoma NOS | 1 |
| Plasmocytoma | 2 |
| Pleomorphic carcinoma | 2 |
| Primary neuroectodermal tumor | 1 |
| Rhabdoid tumor NOS | 1 |
| Sarcoma | 4 |
| Squamous cell carcinoma, HN | 1 |
| Undifferentiated carcinoma | 2 |
| Undifferentiated tumor NOS | 2 |
| Urothelial | 4 |
|  | 50 |

GI, gastrointestinal; HN, head and neck; NOS, not otherwise specified

**Appendix 3** – Full list of included studies

| **Author** | **Year** | **Country** | **Number of kidney recipients** | **Number of transmissions** | **Notes** |
| --- | --- | --- | --- | --- | --- |
| ***Case reports*** |  |  |  |  |  |
| Aigelsreiter [1] | 2009 | Austria | 1 | 1 |  |
| Alhuraiji [2] | 2014 | Saudi Arabia | 1 | 1 |  |
| Baird [3] | 1975 | UK | 1 | 1 |  |
| Barrou [4] | 2001 | France | 1 | 1 | Also heart |
| Baudoux [5] | 2017 | Belgium | 2 | 1 |  |
| Bodvarsson [6] | 2001 | USA | 1 | 1 |  |
| Bosmans [7] | 1997 | Belgium | 1 | 1 |  |
| Boyle [8] | 2017 | USA | 1 | 1 | Also one liver with no data |
| Cankovic [9] | 2006 | USA | 1 | 1 | Note on a liver case |
| Cassol [10] | 2019 | USA | 2 | 1 |  |
| Champion [11] | 2017 | France | 1 | 1 |  |
| Chen [12] | 2012 | USA | 1 | 1 | Also liver with no data |
| Cheung [13] | 1998 | China | 2 | 1 |  |
| Christensen [14] | 2015 | Denmark | 1 | 1 |  |
| Cibeira [15] | 2003 | Spain | 1 | 1 | Kidney-pancreas |
| Cogny [16] | 1995 | France | 1 | 1 |  |
| Detry [17] | 2005 | Belgium | 2 | 1 | Also liver |
| Doutrelepont [18] | 1995 | Belgium | 1 | 1 |  |
| Ferreira [19] | 2010 | Brazil | 2 | 1 | Also on liver with no data |
| Foltys [20] | 2009 | Germany | 2 | 1 | Also liver |
| Fritsche [21] | 1999 | Germany | 1 | 1 |  |
| Fujiwara [22] | 2019 | Japan | 1 | 1 | Living donor |
| Gerstenkorn [23] | 2003 | Germany | 1 | 1 | Also liver |
| Girsberger [24] | 2013 | Switzerland | 1 | 1 |  |
| Gokel [25] | 1977 | Germany | 1 | 1 |  |
| Greco [26] | 2005 | USA | 1 | 1 | Living donor |
| Grey [27] | 2000 | UK | 1 | 1 |  |
| Heinz-Peer [28] | 1994 | Austria | 1 | 1 |  |
| Hernandez Rivera [29] | 2018 | Mexico | 1 | 1 | Living donor |
| Harvey [30] | 1981 | UK | 1 | 1 |  |
| Hjelle [31] | 1988 | USA | 1 | 1 |  |
| Homburg [32] | 1988 | Germany | 1 | 1 |  |
| Huurman [33] | 2008 | Netherlands | 1 | 1 |  |
| Jaillard [34] | 2015 | France | 1 | 1 | Living donor |
| Jeremy [35] | 1972 | Australia | 2 | 1 |  |
| Kim [36] | 2009 | USA | 2 | 1 | Note on a liver case |
| Kriegshauser [37] | 2006 | USA | 1 | 1 | Living donor |
| Kowal [38] | 2008 | Poland | 1 | 1 |  |
| Kunisch-Hoppe [39] | 1998 | Germany | 1 | 1 |  |
| Lappin [40] | 1999 | UK | 1 | 1 |  |
| Lotan [41] | 1995 | Israel | 1 | 1 |  |
| Martin [42] | 1965 | USA | 1 | 1 |  |
| McCanty [43] | 1989 | USA | 1 | 1 |  |
| McIntosh [44] | 1965 | USA | 1 | 1 |  |
| Meduri [45] | 1991 | France | 1 | 1 |  |
| Michel Ortega [46] | 2016 | USA | 1 | 1 | Living donor |
| Milton [47] | 2006 | Australia | 2 | 1 |  |
| Mocelin [48] | 1975 | Brazil | 1 | 1 |  |
| Morath [49] | 2005 | Germany | 2 | 1 |  |
| Muiznieks [50] | 1968 | USA | 1 | 1 |  |
| Neipp [51] | 2006 | Germany | 1 | 1 | Living donor |
| Ng [52] | 2000 | USA | 1 | 1 | Also liver |
| Packer [53] | 1988 | UK | 1 | 1 |  |
| Peri [54] | 2006 | USA | 1 | 1 |  |
| Peters [55] | 1978 | USA | 1 | 1 |  |
| Phelan [56] | 2010 | Ireland | 2 | 1 |  |
| Renoult [57] | 1995 | France | 1 | 1 |  |
| Schlieper [58] | 2006 | Germany | 2 | 1 |  |
| Schütt [59] | 1993 | Germany | 2 | 1 |  |
| Shustik [60] | 1995 | Canada | 1 | 1 |  |
| Tunner [61] | 1971 | USA | 1 | 1 |  |
| Val-bernal [62] | 1993 | Spain | 1 | 1 | Also in Ruiz 1993 |
| Vincent [63] | 1994 | France | 1 | 1 |  |
| Williams [64] | 2012 | USA | 1 | 1 |  |
| Wilson [65] | 1968 | USA | 1 | 1 |  |
| Wilson [66] | 1992 | USA | 1 | 1 |  |
| Wong [67] | 2007 | UK | 1 | 1 | Note on a pancreas transplant case; also liver |
| Xiong [68] | 2017 | China | 1 | 1 | Two kidneys in the same recipient |
| Yaich [69] | 2011 | Tunisia | 2 | 1 |  |
| Zavos [70] | 2004 | Greece | 1 | 1 |  |
| Zhao [71] | 2012 | USA | 1 | 1 | Multivisceral transplant |
| Zukoski [72] | 1970 | USA | 1 | 1 |  |
|  |  |  |  |  |  |
| ***Case series*** |  |  |  |  |  |
| Baehner [73] | 2000 | USA | 2 | 2 | Also liver |
| Baquero [74] | 1988 | USA | 2 | 2 | Also liver and heart |
| Barozzi [75] | 2003 | Italy | 8 | 5 |  |
| Barnes [76] | 1976 | UK | 2 | 1 |  |
| Beckingham [77] | 1994 | UK | 2 | 2 |  |
| Bentdal [78] | 1994 | Norway | 2 | 2 |  |
| Braun-Parvez [79] | 2010 | France | 2 | 2 | Kidney-pancreas; also liver and heart |
| Caillard [80] | 2005 | France | 2 | 2 |  |
| Colquhoun [81] | 1994 | USA | 45 | 2 |  |
| Conlon [82] | 1995 | USA | 2 | 2 |  |
| Detry [83] | 1994 | France | 6 | 1 |  |
| Dziewanowski [84] | 2014 | Poland | 2 | 2 |  |
| Eccher [85] | 2018 | Italy | 6 | 6 | Another case known after publication |
| Elder [86] | 1997 | Australia | 2 | 2 | Also liver and heart |
| Fairman [87] | 1980 | USA | 2 | 2 |  |
| Felldin [88] | 2016 | Sweden | 4 | 4 | Also two liver recipients |
| Forbes [89] | 1981 | UK | 2 | 2 |  |
| Georgieva [90] | 2016 | Belgium | 3 | 3 |  |
| Göbel [91] | 2007 | Germany | 2 | 2 |  |
| Harbell [92] | 2008 | USA | 2 | 2 | Also liver and pancreas |
| Heinz-Peer [93] | 1995 | Austria | 2 | 2 | One already in Heinz-Peer 1994 |
| Herzig [94] | 2000 | Australia | 2 | 2 |  |
| Jones [95] | 1994 | Canada | 2 | 2 |  |
| Königsrainer [96] | 1993 | Austria | 2 | 2 |  |
| Kooistra [97] | 1995 | Netherlands | 2 | 2 |  |
| Krapp [98] | 2005 | Germany | 2 | 2 |  |
| Lanari [99] | 1972 | Argentina | 4 | 4 |  |
| Larson [100] | 1996 | USA | 4 | 1 |  |
| Lefrancois [101] | 1987 | France | 2 | 2 | Also heart |
| Lipshutz [102] | 2009 | USA | 2 | 2 |  |
| Llamas [103] | 2009 | Spain | 2 | 2 |  |
| MacKie [104] | 2003 | UK | 2 | 2 |  |
| Maclean [105] | 1965 | Canada | 19 | 1 |  |
| Marsh [106] | 1987 | USA | 2 | 2 |  |
| Matser [107] | 2018 | Netherlands | 2 | 2 | Also liver and double lungs |
| Morris-Stiff [108] | 2004 | UK | 2 | 2 | Also liver |
| Olagne [109] | 2011 | France | 14 | 14 |  |
| Palanisamy [110] | 2015 | USA | 2 | 2 |  |
| Pandanaboyana [111] | 2016 | UK | 2 | 1 |  |
| Penn [112] | 1996 | USA | 20 | 14 |  |
| Robin [113] | 2016 | USA | 8 | 5 | Two living donors |
| Ruiz [114] | 1993 | Spain | 2 | 2 | Case 1 in Val-bernal 1993 |
| Schattenkirchner [115] | 1995 | Germany | 2 | 2 | Same cases as in Barth 1997 |
| Schmidt [116] | 1995 | Germany | 868 | 1 |  |
| Schmitt [117] | 1998 | Germany | 2 | 2 |  |
| Singh [118] | 2019 | USA | 2 | 2 | Other cornea recipient |
| Stephens [119] | 2000 | USA | 2 | 2 | Also liver and heart |
| Thoning [120] | 2013 | Denmark | 2 | 2 | Also liver and lung |
| Wilson [121] | 1975 | USA | 47 | 15 | 12 cases reported elsewhere |
| Yamaçake [122] | 2015 | Brazil | 2 | 2 |  |
|  |  |  |  |  |  |
| ***Registries*** |  |  |  |  |  |
| Birkenland [123] | 2002 | Denmark | 626 | 1 |  |
| Buell [124] | 2003 | USA | NA | 2 | Also liver |
| Desai [125] | 2012 | UK | 25 | 12 |  |
| Garrido [126] | 2008 | Spain | 64 | 6 | Also liver and lung |
| Ison [127] | 2009 | USA | 2 | 2 |  |
| Kauffman [128] | 2002 | USA | NA | 7 | Also liver and heart; some cases already reported |

**References**

1. Aigelsreiter A, Siegler P, Pilcher M, et al (2009) Transmission of metastasizing high-grade hepatocellular carcinoma from a donor with liver cirrhosis. Virchows Arch 455:1–482. https://doi.org/10.1007/s00428-009-0805-z

2. Alhuraiji A, Chebbo W, El-Gohary G, et al (2015) Donor-derived extramedullary acute promyelocytic leukemia post kidney transplant. Ann Hematol 94:505–507. https://doi.org/10.1007/s00277-014-2200-3

3. Baird RN, White HJ, Tribe CR (1975) Renal carcinoma in a cadaver kidney graft donor. BMJ 2:371–371. https://doi.org/10.1136/bmj.2.5967.371

4. Barrou B, Bitker MO, Delcourt A, et al (2001) Fate of a renal tubulopapillary adenoma transmitted by an organ donor. Transplantation 72:540–1. http://www.ncbi.nlm.nih.gov/pubmed/11502993

5. Baudoux TER, Gastaldello K, Rorive S, et al (2017) Donor Cancer Transmission in Kidney Transplantation. Kidney Int Reports 2:134–137. https://doi.org/10.1016/j.ekir.2016.09.057

6. Bodvarsson S, Burlingham W, Kusaka S, et al (2001) Donor-derived small cell lung carcinoma in a kidney transplant recipient. Cancer 92:2429–2434. https://doi.org/10.1002/1097-0142(20011101)92:9<2429::AID-CNCR1592>3.0.CO;2-G

7. Bosmans J-L, Ysebaert D, De Cock AM, et al (1997) Interferon-alpha and the cure of metastasis of a malignant meningioma in a kidney allograft recipient: A case report. Transplant Proc 29:838. https://doi.org/10.1016/S0041-1345(96)00156-X

8. Boyle SM, Ali N, Olszanski AJ, et al (2017) Donor-Derived Metastatic Melanoma and Checkpoint Inhibition. Transplant Proc 49:1551–1554. https://doi.org/10.1016/j.transproceed.2017.06.007

9. Cankovic M, Linden MD, Zarbo RJ (2006) Use of microsatellite analysis in detection of tumor lineage as a cause of death in a liver transplant patient. Arch Pathol Lab Med 130:529–532. https://doi.org/10.1043/1543-2165(2006)130[529:UOMAID]2.0.CO;2

10. Cassol CA, Hod‐Dvorai R, Hubbell C, et al (2019) Donor‐derived Philadelphia chromosome‐positive B cell lymphoblastic leukemia presenting with renal allograft involvement in the first year posttransplant. Am J Transplant 19:956–957. https://doi.org/10.1111/ajt.15117

11. Champion L, Culine S, Desgranchamps F, et al (2017) Metastatic Renal Cell Carcinoma in a Renal Allograft: A Sustained Complete Remission After Stimulated Rejection. Am J Transplant 17:1125–1128. https://doi.org/10.1111/ajt.14151

12. Chen KT, Olszanski A, Farma JM (2012) Donor Transmission of Melanoma Following Renal Transplant. Case Rep Transplant 2012:1–3. https://doi.org/10.1155/2012/764019

13. Cheung AN, Chan AC, Chung LP, et al (1998) Post-transplantation lymphoproliferative disorder of donor origin in a sex-mismatched renal allograft as proven by chromosome in situ hybridization. Mod Pathol 11:99–102. http://www.ncbi.nlm.nih.gov/pubmed/9556430

14. Christensen SF, Hansen JM (2015) Donor Kidney With Renal Cell Carcinoma Successfully Treated With Radiofrequency Ablation: A Case Report. Transplant Proc 47:3031–3033. https://doi.org/10.1016/j.transproceed.2015.10.039

15. Cibeira MT, Lopez-Guillermo A, Colomer D, et al (2003) Diffuse large B-cell lymphoma arising from donor lymphoid cells after renal and pancreatic transplantation. Ann Hematol 82:131–135. https://doi.org/10.1007/s00277-002-0591-z

16. Cogny-Van Weydevelt F, Harry P, Bourree Y, Riberi P (1995) Graft of a trophoblastic neoplasia by a kidney transplant. Transplant Proc 27:1791–2. http://www.ncbi.nlm.nih.gov/pubmed/7725507

17. Detry O, De Roover A, de Leval L, et al (2005) Transmission of an undiagnosed sarcoma to recipients of kidney and liver grafts procured in a non-heart beating donor. Liver Transplant 11:696–699. https://doi.org/10.1002/lt.20457

18. Doutrelepont JM, Mat O, Abramowicz D, et al (1995) Inadvertent transfer of choriocarcinoma with renal transplantation: characteristics of the donor-recipient pairs. Transplant Proc 27:1789–90. http://www.ncbi.nlm.nih.gov/pubmed/7725506

19. Ferreira GF, de Oliveira RA, Jorge LB, et al (2010) Urothelial carcinoma transmission via kidney transplantation. Nephrol Dial Transplant 25:641–643. https://doi.org/10.1093/ndt/gfp612

20. Foltys D, Linkermann A, Heumann A, et al (2009) Organ Recipients Suffering From Undifferentiated Neuroendocrine Small-Cell Carcinoma of Donor Origin: A Case Report. Transplant Proc 41:2639–2642. https://doi.org/10.1016/j.transproceed.2009.06.026

21. Fritsche L, Budde K, Rogalla P, et al (1999) Successful living related kidney transplantation despite renal angiomyolipoma in situ. J Urol 162:480–1. https://doi.org/10411061

22. Fujiwara S, Ikeda T, Morita K, et al (2019) Multiple myeloma derived from a kidney transplant donor who also developed myeloma after kidney donation. Am J Transplant 19:2374–2377. https://doi.org/10.1111/ajt.15373

23. Gerstenkorn C, Thomusch O (2003) Transmission of a pancreatic adenocarcinoma to a renal transplant recipient. Clin Transplant 17:473–476. https://doi.org/10.1034/j.1399-0012.2003.00072.x

24. Girsberger S, Wehmeier C, Amico P, et al (2013) Donor-derived acute myeloid leukemia in a kidney transplant recipient. Blood 122:298–300. https://doi.org/10.1182/blood-2013-04-497875

25. Gokel JM, Rjosk HK, Meister P, et al (1977) Metastatic choriocarcinoma transplanted with cadaver kidney.A case report. Cancer 39:1317–1321. https://doi.org/10.1002/1097-0142(197703)39:3<1317::AID-CNCR2820390345>3.0.CO;2-A

26. Greco AJ, Baluarte JH, Meyers KEC, et al (2005) Chromophobe Renal Cell Carcinoma in a Pediatric Living-Related Kidney Transplant Recipient. Am J Kidney Dis 45:e105–e108. https://doi.org/10.1053/j.ajkd.2005.03.012

27. Grey M, Townsend N, Lappin D, et al (2000) IgA myeloma of donor origin arising 7 years after allogeneic renal transplant. Br J Haematol 108:592–594. https://doi.org/10.1046/j.1365-2141.2000.01913.x

28. Heinz-Peer G, Helbich T, Nöttling B, et al (1994) Renal cell carcinoma in an allograft kidney transplant. Transplantation 57:475–478. https://doi.org/10.1097/00007890-199402150-00033

29. Hernández-Rivera JCH, Pérez-López MJ, Cardona-Chávez JG, et al [Non-Hodgkin lymphoma. Incidental finding in a renal donor, 10 years after the evolution in recipient]. Rev Med Inst Mex Seguro Soc 56:112–115. http://www.ncbi.nlm.nih.gov/pubmed/29368904

30. Harvey L, Fox M (1981) Transferral of malignancy as a complication of organ transplantation: an insuperable problem? J Clin Pathol 34:116–122. https://doi.org/10.1136/jcp.34.2.116

31. Hjelle B, Evans-Holm M, Benedict Yen TS, et al (1989) A poorly differentiated lymphoma of donor origin in a renal allograft recipient. Transplantation 47:945–948. https://doi.org/10.1097/00007890-198906000-00005

32. Homburg A, Kindler J, Hofstädter F, et al (1988) Regression of an adenocarcinoma transmitted by a cadaver kidney graft. Transplantation 46:777–9. http://www.ncbi.nlm.nih.gov/pubmed/3057702

33. Huurman VAL, Baranski AG, Groeneveld JHM, et al (2008) Transfer of ureteral carcinoma in a transplanted kidney presenting by early stenosis of the proximal ureter. Clin Transplant 22:847–850. https://doi.org/10.1111/j.1399-0012.2008.00889.x

34. Jaillard A, Baillet C, Béron A, et al (2016) FDG PET/CT allowing detection and follow-up of tumor cell transplantation. Ann Nucl Med 30:250–254. https://doi.org/10.1007/s12149-015-1051-x

35. Jeremy D, Farnsworth RH, Robertson MR, et al (1972) Transplantation of malignant melanoma with cadaver kidney. Transplantation 13:619. https://doi.org/10.1097/00007890-197206000-00015

36. Kim JK, Carmody IC, Cohen AJ, Loss GE Donor transmission of malignant melanoma to a liver graft recipient: case report and literature review. Clin Transplant 23:571–4. https://doi.org/10.1111/j.1399-0012.2008.00928.x

37. Kriegshauser JS, Weidenfeld PL, Wochos DN, Williams JW (2006) Auto-Rejection of Renal Donor-Origin Metastatic Melanoma. Radiol Case Reports 1:149–153. https://doi.org/10.2484/rcr.v1i4.44

38. Kowal M, Hus M, Dmoszynska A, et al (2008) Acute T Cell Lymphoblastic Leukemia in the Recipient of a Renal Transplant from a Donor with Malignant Lymphoma. Acta Haematol 119:187–189. https://doi.org/10.1159/000137944

39. Kunisch-Hoppe M, Hoppe M, Bohle RM, et al (1998) Metastatic RCC arising in a transplant kidney. Eur Radiol 8:1441–1443. https://doi.org/10.1007/s003300050570

40. Lappin D (1999) Angiomyolipoma in a transplanted kidney. Nephrol Dial Transplant 14:1574–1575. https://doi.org/10.1093/ndt/14.6.1574

41. Lotan D, Laufer J (1995) Metastatic renal carcinoma in a pediatric recipient of an adult cadaveric donor kidney. Am J Kidney Dis 26:960–962. https://doi.org/10.1016/0272-6386(95)90063-2

42. Martin DC (1965) Cadaveric Renal Homotransplantation With Inadvertent Transplantation of Carcinoma. JAMA J Am Med Assoc 192:752. https://doi.org/10.1001/jama.1965.03080220016003

43. McCanty TC, Jonsson J, Khawand N, et al (1989) Transferral of a malignancy with a transplanted kidney. Transplantation 48:877–878. https://doi.org/10.1097/00007890-198911000-00033

44. McIntosh DA (1965) Homotransplantation of a Cadaver Neoplasm and a Renal Homograft. JAMA J Am Med Assoc 192:1171. https://doi.org/10.1001/jama.1965.03080260059024

45. Meduri G, Fromentin L, Vieillefond A, Fries D (1991) Donor-related non-Hodgkin’s lymphoma in a renal allograft recipient. Transplant Proc 23:2649. http://www.ncbi.nlm.nih.gov/pubmed/1926517

46. Michel Ortega RM, Wolff DJ, Schandl CA, Drabkin HA (2016) Urothelial carcinoma of donor origin in a kidney transplant patient. J Immunother Cancer 4:63. https://doi.org/10.1186/s40425-016-0167-4

47. Milton CA, Barbara J, Cooper J, et al (2006) The transmission of donor-derived malignant melanoma to a renal allograft recipient. Clin Transplant 20:547–550. https://doi.org/10.1111/j.1399-0012.2006.00514.x

48. Mocelin AJ, Brandina L (1975) Inadvertent transplant of a malignancy. Transplantation 19:430. https://doi.org/10.1097/00007890-197505000-00011

49. Morath C, Rohmeiss P, Schwenger V, et al (2005) Transmission of Donor-Derived Small-Cell Carcinoma Cells by a Nontumor-Bearing Allograft. Transplantation 80:540–542. https://doi.org/10.1097/01.tp.0000168489.71242.fd

50. Muiznieks HW, Berg JW, Lawrence W, Randall HT (1968) Suitability of donor kidneys from patients with cancer. Surgery 64:871–7. http://www.ncbi.nlm.nih.gov/pubmed/4879937

51. Neipp M, Schwarz A, Pertschy S, et al (2006) Accidental transplantation of a kidney with a cystic renal cell carcinoma following living donation: management and 1 yr follow-up. Clin Transplant 20:147–150. https://doi.org/10.1111/j.1399-0012.2005.00455.x

52. Ng IOL, Shek TWH, Thung SN, et al (2000) Microsatellite Analysis in Post-Transplantation Lymphoproliferative Disorder to Determine Donor/Recipient Origin. Mod Pathol 13:1180–1185. https://doi.org/10.1038/modpathol.3880218

53. Packer GJ, Ross WB, Salaman JR (1988) Inadvertent Transplantation of a Renal Carcinoma. Br J Urol 62:614–615. https://doi.org/10.1111/j.1464-410X.1988.tb04441.x

54. Peri N, Kussick S, Bakthavatsalam R, et al (2006) Postrenal Transplant Non-EBV Multiple Myeloma of Donor Origin. Am J Transplant 6:419–422. https://doi.org/10.1111/j.1600-6143.2005.01170.x

55. Peters MS, Stuard ID (1978) Metastatic malignant melanoma transplanted via a renal homograft.A case report. Cancer 41:2426–2430. https://doi.org/10.1002/1097-0142(197806)41:6<2426::AID-CNCR2820410649>3.0.CO;2-0

56. Phelan PJ, Murphy RKJ, Farrell M, et al (2010) EBV-positive B cell cerebral lymphoma 12 years after sex-mismatched kidney transplantation: post-transplant lymphoproliferative disorder or donor-derived lymphoma? Nephrol Dial Transplant 25:2032–5. https://doi.org/10.1093/ndt/gfq170

57. Renoult E, Aymard B, Grégoire M-J, et al (1995) Epstein-Barr virus lymphoproliferative disease of donor origin after kidney transplantation: A case report. Am J Kidney Dis 26:84–87. https://doi.org/10.1016/0272-6386(95)90159-0

58. Schlieper G, Kurschat C, Donner A, et al (2006) Hodgkin Disease–Like Posttransplantation Lymphoproliferative Disorder of Donor Origin in a Renal Allograft Recipient. Am J Kidney Dis 47:e37–e41. https://doi.org/10.1053/j.ajkd.2005.11.022

59. Schütt G, Engemann R, Gassel HJ, et al (1993) Donor-transmitted non-Hodgkin’s lymphoma after renal transplantation--a case report. Transplant Proc 25:2131–2. http://www.ncbi.nlm.nih.gov/pubmed/8470292

60. Shustik C, Jamison BM, Alfieri C, et al (1995) A solitary plasmacytoma of donor origin arising 14 years after kidney allotransplantation. Br J Haematol 91:167–168. https://doi.org/10.1111/j.1365-2141.1995.tb05263.x

61. Tunner WS, Goldsmith EI, Whitsell JC (1971) Human Homotransplantation of Normal and Neoplastic Tissue from the Same Organ. J Urol 105:18–20. https://doi.org/10.1016/S0022-5347(17)61451-4

62. Val-Bernal F, Ruiz JC, Cotorruelo JG, Arias M (1993) Glioblastoma multiforme of donor origin after renal transplantation: Report of a case. Hum Pathol 24:1256–1259. https://doi.org/10.1016/0046-8177(93)90224-5

63. Vincent F, Levy V, Bensousan TA, et al (1994) “Spontaneous” regressions of a metastatic adenocarcinoma transmitted by a cadaver kidney graft: Support for immunotherapy? Cancer Immunol Immunother 39:205–206. https://doi.org/10.1007/BF01533388

64. Williams T, Aljitawi OS, Moussa R, et al (2012) First case of donor transmitted non-leukemic promyelocytic sarcoma. Leuk Lymphoma 53:2530–2534. https://doi.org/10.3109/10428194.2012.695360

65. Wilson RE, Hager EB, Hampers CL, et al (1968) Immunologic Rejection of Human Cancer Transplanted with a Renal Allograft. N Engl J Med 278:479–483. https://doi.org/10.1056/NEJM196802292780904

66. Wilson LJ, Horvat RT, Tilzer L, et al (1992) Identification of donor melanoma in a renal transplant recipient. Diagn Mol Pathol 1:266–71. http://www.ncbi.nlm.nih.gov/pubmed/1364174

67. Wong C, Hold P, Mohteshamzadeh M, et al (2007) Occult Donor Malignancy in Pancreas Transplantation. Ren Fail 29:243–244. https://doi.org/10.1080/08860220601100536

68. Xiong J, Su T, Zhu P, et al (2017) Malignant rhabdoid tumor in the renal allograft of an adult transplant recipient: a unique case of a rare tumor. Diagn Pathol 12:86. https://doi.org/10.1186/s13000-017-0677-5

69. Yaich S, El’Aoud N, Zaghdane S, et al (2011) Primary Adenocarcinoma in a Kidney Allograft: A Case Report and Review of the Literature. Transplant Proc 43:660–662. https://doi.org/10.1016/j.transproceed.2011.01.074

70. Zavos G, Papaconstantinou I, Chrisostomidis C, Kostakis A (2004) Metastatic melanoma within a transplanted kidney: a case report. Transplant Proc 36:1411–1412. https://doi.org/10.1016/j.transproceed.2004.05.010

71. Zhao P, Strohl A, Gonzalez C, et al (2012) Donor transmission of pineoblastoma in a two-yr-old male recipient of a multivisceral transplant: A case report. Pediatr Transplant 16:E110–E114. https://doi.org/10.1111/j.1399-3046.2010.01463.x

72. Zukoski CF, Killen DA, Ginn E, et al (1970) Transplanted carcinoma in an immunosuppressed patient. Transplantation 9:71–74. https://doi.org/10.1097/00007890-197001000-00021

73. Baehner R, Magrane G, Balassanian R, et al (2000) Donor origin of neuroendocrine carcinoma in 2 transplant patients determined by molecular cytogenetics. Hum Pathol 31:1425–1429. https://doi.org/10.1016/S0046-8177(00)80015-5

74. Baquero A, Foote J, Kottle S, et al (1988) Inadvertent transplantation of choriocarcinoma into four recipients. Transplant Proc 20:98–100. http://www.ncbi.nlm.nih.gov/pubmed/3278469

75. Barozzi P, Luppi M, Facchetti F, et al (2003) Post-transplant Kaposi sarcoma originates from the seeding of donor-derived progenitors. Nat Med 9:554–561. https://doi.org/10.1038/nm862

76. Barnes AD, Fox M (1976) Transplantation of tumour with a kidney graft. BMJ 1:1442–1444. https://doi.org/10.1136/bmj.1.6023.1442

77. Beckingham IJ, O’Rourke JS, Bishop MC, et al (1994) The use of DNA typing to clarify the origin of metastatic carcinoma after renal transplantation. Transpl Int 7:379–381. https://doi.org/10.1007/BF00336716

78. Bentdal OH, Brekke IB, Lien B, et al (1994) Rapid development of cancer in both kidney grafts after transplantation from a donor with undiagnosed malignant disease. Transplant Proc 26:1763 http://www.ncbi.nlm.nih.gov/pubmed/8030123

79. Braun-Parvez L, Charlin E, Caillard S, et al (2010) Gestational Choriocarcinoma Transmission Following Multiorgan Donation. Am J Transplant 10:2541–2546. https://doi.org/10.1111/j.1600-6143.2010.03275.x

80. Caillard S, Pencreach E, Braun L, et al (2005) Simultaneous Development of Lymphoma in Recipients of Renal Transplants from a Single Donor: Donor Origin Confirmed by Human Leukocyte Antigen Staining and Microsatellite Analysis. Transplantation 79:79–84. https://doi.org/10.1097/01.TP.0000146385.40108.2B

81. Colquhoun SD, Robert ME, Shaked A, et al (1994) Transmission of CNS malignancy by organ transplantation. Transplantation 57:970–4. http://www.ncbi.nlm.nih.gov/pubmed/8154048

82. Conlon PJ, Smith SR (1995) Transmission of cancer with cadaveric donor organs. J Am Soc Nephrol 6:54–60. http://www.ncbi.nlm.nih.gov/pubmed/7579070

83. Detry O, Detroz B, D’Silva M, Pirenne J (1994) Transplantations de tumeurs malignes insoupçonnées lors de greffes d’organes. Rev. Médicale. http://orbi.ulg.ac.be/handle/2268/26357

84. Dziewanowski K, Drozd R, Parczewski M, Klinke M (2014) Multiorgan transplantation from a deceased donor with intravascular diffuse large B-cell lymphoma: transmission of the disease and results of treatment. Clin Transplant 28:1080–3. https://doi.org/10.1111/ctr.12417

85. Eccher A, Lombardini L, Girolami I, et al (2019) How safe are organs from deceased donors with neoplasia? The results of the Italian Transplantation Network. J Nephrol 32:323–330. https://doi.org/10.1007/s40620-018-00573-z

86. Elder GJ, Hersey P, Branley P (1997) Remission of transplanted melanoma--clinical course and tumour cell characterisation. Clin Transplant 11:565–8. http://www.ncbi.nlm.nih.gov/pubmed/9408685

87. Fairman RM, Grossman RA, Barker CF, Perloff LJ (1980) Inadvertent transplantation of a melanoma. Transplantation 30:328–330. https://doi.org/10.1097/00007890-198011000-00003

88. Felldin M, Ekberg J, Polanska-Tamborek D, et al (2016) Donor Monoclonal Gammopathy May Cause Lymphoproliferative Disorders in Solid Organ Transplant Recipients. Am J Transplant 16:2676–2683. https://doi.org/10.1111/ajt.13804

89. Forbes GB, Goggin MJ, Dische FE, et al (1981) Accidental transplantation of bronchial carcinoma from a cadaver donor to two recipients of renal allografts. J Clin Pathol 34:109–115. https://doi.org/10.1136/jcp.34.2.109

90. Georgieva LA, Gielis EM, Hellemans R, et al (2016) Single-Center Case Series of Donor-Related Malignancies: Rare Cases With Tremendous Impact. Transplant Proc 48:2669–2677. https://doi.org/10.1016/j.transproceed.2016.07.014

91. Göbel H, Gloy J, Neumann J, et al (2007) Donor-Derived Small Cell Lung Carcinoma in a Transplanted Kidney. Transplantation 84:800–802. https://doi.org/10.1097/01.tp.0000281402.55745.e6

92. Harbell JW, Dunn TB, Fauda M, et al (2008) Transmission of anaplastic large cell lymphoma via organ donation after cardiac death. Am J Transplant 8:238–44. https://doi.org/10.1111/j.1600-6143.2007.02033.x

93. Heinz-Peer G, Helbich T, Barnas U, et al (1995) Renal cell carcinomas of donor origin in two kidney transplants from a single donor. Transplantation 59:912–3. http://www.ncbi.nlm.nih.gov/pubmed/7701592

94. Herzig KA, Falk MC, Jonsson JR, et al (2000) Novel surveillance and cure of a donor-transmitted lymphoma in a renal allograft recipient. Transplantation 70:149–52. http://www.ncbi.nlm.nih.gov/pubmed/10919592

95. Jones C, Bleau B, Buskard N, et al (1994) Simultaneous Development of Diffuse Immunoblastic Lymphoma in Recipients of Renal Transplants From a Single Cadaver Donor: Transmission of Epstein-Barr Virus and Triggering by OKT3. Am J Kidney Dis 23:130–134. https://doi.org/10.1016/S0272-6386(12)80823-7

96. Königsrainer A, Steurer W, Schumer J, et al (1993) Transmission of non-Hodgkin’s lymphoma through renal allografts--disastrous result of false diagnosis and inadequate information. Transplant Proc 25:3075–6. http://www.ncbi.nlm.nih.gov/pubmed/8266458

97. Kooistra MP, Vos PF, de Graaf PW, et al (1995) Metastatic renal-cell carcinoma developing in two recipients of renal grafts derived from the same donor. Nephrol Dial Transplant 10:1772–4. http://www.ncbi.nlm.nih.gov/pubmed/8559506

98. Krapp JD, Brauer RB, Matevossian E, et al (2005) Donor transmitted anaplastic carcinoma in a kidney-transplant recipient. Transpl Int 18:1109–1112. https://doi.org/10.1111/j.1432-2277.2005.00178.x

99. Lanari A, Rodŏ JE, Barcat JA, et al [4 cases of donor-cancer development in recipients of a transplanted kidney]. Medicina (B Aires) 32:79–92. http://www.ncbi.nlm.nih.gov/pubmed/4556900

100. Larson RS, Scott MA, McCurley TL, Vnencak-Jones CL (1996) Microsatellite analysis of posttransplant lymphoproliferative disorders: Determination of donor/recipient origin and identification of putative lymphomagenic mechanism. Cancer Res 56:4378–4381. http://www.ncbi.nlm.nih.gov/pubmed/8813129

101. Lefrancois N, Touraine JL, Cantarovich D, et al (1987) Transmission of medulloblastoma from cadaver donor to three organ transplant recipients. Transplant Proc 19:2242 http://www.ncbi.nlm.nih.gov/pubmed/3274501

102. Lipshutz GS, Mihara N, Wong R, et al (2009) Death from Metastatic Donor-Derived Ovarian Cancer in a Male Kidney Transplant Recipient. Am J Transplant 9:428–432. https://doi.org/10.1111/j.1600-6143.2008.02507.x

103. Llamas F, Gallego E, Salinas A, et al (2009) Sarcomatoid renal cell carcinoma in a renal transplant recipient. Transplant Proc 41:4422–4. https://doi.org/10.1016/j.transproceed.2009.08.066

104. MacKie RM, Reid R, Junor B (2003) Fatal Melanoma Transferred in a Donated Kidney 16 Years after Melanoma Surgery. N Engl J Med 348:567–568. https://doi.org/10.1056/NEJM200302063480620

105. MacLean LD (1965) Renal Homotransplantation Using Cadaver Donors. Arch Surg 91:288. https://doi.org/10.1001/archsurg.1965.01320140078012

106. Marsh JW, Esquivel CO, Makowka L, et al (1987) Accidental transplantation of malignant tumor from a donor to multiple recipients. Transplantation 44:449–50. https://doi.org/10.1097/00007890-198709000-00025

107. Matser YAH, Terpstra ML, Nadalin S, et al (2018) Transmission of breast cancer by a single multiorgan donor to 4 transplant recipients. Am J Transplant 18:1810–1814. https://doi.org/10.1111/ajt.14766

108. Morris-Stiff G, Steel A, Savage P, et al (2004) Transmission of donor melanoma to multiple organ transplant recipients. Am J Transplant 4:444–6. http://doi.wiley.com/10.1111/j.1600-6143.2004.00335.x

109. Olagne J, Caillard S, Gaub MP, et al (2011) Post-transplant Lymphoproliferative Disorders: Determination of Donor/Recipient Origin in a Large Cohort of Kidney Recipients. Am J Transplant 11:1260–1269. https://doi.org/10.1111/j.1600-6143.2011.03544.x

110. Palanisamy A, Persad P, Koty PP, et al (2015) Donor-Derived Myeloid Sarcoma in Two Kidney Transplant Recipients from a Single Donor. Case Reports Nephrol 2015:1–5. https://doi.org/10.1155/2015/821346

111. Pandanaboyana S, Longbotham D, Hostert L, et al (2016) Transplantation of liver and kidney from donors with malignancy at the time of donation: an experience from a single centre. Transpl Int 29:73–80. https://doi.org/10.1111/tri.12693

112. Penn I (1996) Malignant melanoma in organ allograft recipients. Transplantation 61:274–278. https://doi.org/10.1097/00007890-199601270-00019

113. Robin AJ, Cohen EP, Chongkrairatanakul T, et al (2016) A single center’s approach to discriminating donor versus host origin of renal neoplasia in the allograft kidney. Ann Diagn Pathol 23:32–34. https://doi.org/10.1016/j.anndiagpath.2016.05.004

114. Ruiz JC, Cotorruelo JG, Tudela V, et al (1993) Transmission of glioblastoma multiforme to two kidney transplant recipients from the same donor in the absence of ventricular shunt. Transplantation 55:682–3. http://www.ncbi.nlm.nih.gov/pubmed/8384384

115. Schattenkirchner S, Ciré K, Krug B, et al (1995) Transmission of a malignant melanoma by allogenic kidney transplantation. Z Hautkr 70:77–79.

116. Schmidt R, Stippel D, Krings F, Pollok M (1995) Malignancies of the genito-urinary system following renal transplantation. Br J Urol 75:572–577. https://doi.org/10.1111/j.1464-410X.1995.tb07409.x

117. Schmitt C, Ciré K, Schattenkirchner S, et al (1998) Highly sensitive DNA typing for detecting tumors transmitted by transplantation. Transpl Int 11:382–386. https://doi.org/10.1007/s001470050162

118. Singh P, Pandey D, Rovin B, et al (2019) Successful Treatment and Five Years of Disease-free Survival in a Donor Transmitted Metastatic Melanoma with Ipilimumab Therapy. Cureus. https://doi.org/10.7759/cureus.4658

119. Stephens JK, Everson GT, Elliott CL, et al (2000) Fatal transfer of malignant melanoma from multiorgan donor to four allograft recipients. Transplantation 70:232–6. http://www.ncbi.nlm.nih.gov/pubmed/10919612

120. Thoning J, Liu Y, Bistrup C, et al (2013) Transmission of Angiosarcomas From a Common Multiorgan Donor to Four Transplant Recipients. Am J Transplant 13:167–173. https://doi.org/10.1111/j.1600-6143.2012.04301.x

121. Wilson RE, Penn I (1975) Fate of tumors transplanted with a renal allograft. Transplant Proc 7:327–31. http://www.ncbi.nlm.nih.gov/pubmed/236612

122. Yamaçake KGR, Antonopoulos IM, Piovesan AC, et al (2015) Donor Transmission Intestinal Carcinoma After Kidney Transplantation: Case Report. Transplant Proc 47:827–830. https://doi.org/10.1016/j.transproceed.2014.12.020

123. Birkeland SA, Storm HH (2002) Risk for tumor and other disease transmission by transplantation: A population-based study of unrecognized malignancies and other diseases in organ donors. Transplantation 74:1409–1413. https://doi.org/10.1097/00007890-200211270-00012

124. Buell JF, Trofe J, Sethuraman G, et al (2003) Donors with central nervous system malignancies: are they truly safe? Transplantation 76:340–3. https://doi.org/10.1097/01.TP.0000076094.64973.D8

125. Desai R, Collett D, Watson CJ, et al (2012) Cancer Transmission From Organ Donors—Unavoidable But Low Risk. Transplant J 94:1200–1207. https://doi.org/10.1097/TP.0b013e318272df41

126. Garrido G, Matesanz R (2008) The Spanish National Transplant Organization (ONT) Tumor Registry. Transplantation 85:S61–S63. https://doi.org/10.1097/TP.0b013e31816c2f55

127. Ison MG, Hager J, Blumberg E, et al (2009) Donor-derived disease transmission events in the United States: data reviewed by the OPTN/UNOS Disease Transmission Advisory Committee. Am J Transplant 9:1929–35. https://doi.org/10.1111/j.1600-6143.2009.02700.x

128. Myron Kauffman H, McBride MA, Cherikh WS, et al (2002) Transplant tumor registry: donor related malignancies. Transplantation 74:358–362. https://doi.org/10.1097/00007890-200208150-00011
